# Transcriptomic data sets for *Novosphingobium aromaticivorans* DSM12444 and a ΔSARO_RS14285 mutant grown in the presence of glucose and either protocatechuic, vanillic, syringic, or 4-coumaric acid

**DOI:** 10.1128/mra.00264-25

**Published:** 2025-07-31

**Authors:** Laura Rodriguez-Castro, Kevin S. Myers, Daniel R. Noguera, Timothy J. Donohue

**Affiliations:** 1 DOE Great Lakes Bioenergy Research Center, Universities of Wisconsin 5229 , Madison, Wisconsin, USA; 2 Wisconsin Energy Institute, University of Wisconsin 732059, Madison, Wisconsin, USA; 3 Department of Civil and Environmental Engineering, Universities of Wisconsin 5229 , Madison, Wisconsin, USA; 4 Department of Bacteriology, Universities of Wisconsin 5229 , Madison, Wisconsin, USA; University of Strathclyde, Glasgow, United Kingdom

**Keywords:** transcriptomics, aromatics, *Novosphingobium aromaticivorans*

## Abstract

The SARO_RS14285 gene, encoding a transcription factor, was deleted in *Novosphingobium aromaticivorans* DSM12444. The transcriptomes of the parent and ΔSARO_RS14285 strains were determined when grown in medium containing glucose with or without protocatechuic, vanillic, syringic, or 4-coumaric acid. We present the raw RNA sequencing data obtained from these cultures.

## ANNOUNCEMENT

Several cellular processes are regulated by transcription factors (TFs) including aromatic compound degradation pathways (1). *Novosphingobium aromaticivorans* DSM12444 is an Alphaproteobacterial species known to metabolize aromatic compounds (2–7). In the *N. aromaticivorans* DSM12444 genome, more than 20 TFs that could be involved in aromatic catabolism were identified (8). *N. aromaticivorans* DSM12444 can be engineered to funnel aromatic compounds found in lignocellulosic biomass into the bioplastic precursor 2-pyrone-4,6-dicarboxylic acid *via* the characterized *meta*-cleavage pathway (2). Immediately upstream of genes encoding known or predicted enzymes to function in aromatic metabolism (SARO_RS14260 to SARO_RS14300) is one gene encoding a predicted LysR-type TF (SARO_RS14285). To determine the role of SARO_RS14285 in *N. aromaticivorans* DSM12444, a mutant containing an in-frame deletion of the gene for this TF was constructed in the Δ*sacB* parent strain (7) by homologous recombination using previously described methods (6, 7, 9). *N. aromaticivorans* DSM12444 was isolated from a polyaromatic hydrocarbon-contaminated site by Fredrickson *et al*. (10). The *sacB* gene (SARO_RS09410, formerly Saro_1879) was deleted previously from *N. aromaticivorans* DSM12444 (7), and the mutant strain was stored at −80°C to allow the use of *sacB*-suicide plasmids for genetic modifications. To identify the role of this TF, a transcriptomic analysis was performed with the parent and mutant strains cultured in glucose alone or glucose plus protocatechuic acid (PCA), vanillic acid (VA), syringic acid (SA), or 4-coumaric acid (4-CA). The ∆*sacB* parent strain *N. aromaticivorans* (DSM12444Δ09410) and ∆SARO_RS14285 mutant strain (DSM12444Δ09410Δ14285) were cultured aerobically at 30°C in standard mineral base (SMB) medium (7) supplemented with 10 mM glucose and 5 mM of an aromatic compound (PCA, 4-CA, VA, or SA) until mid-log phase. The cells were pelleted, frozen in a dry ice bath, and stored at −80°C. They were later thawed, lysed, and RNA was extracted using a hot acid phenol:chloroform extraction, as previously described (11). The samples were treated with RNase-free DNase, and RNA was purified using the RNeasy Kit (Qiagen, Hilden, Germany).

RNA-seq library preparation and sequencing was performed by the Joint Genome Institute (JGI) using standard protocols. rRNA in the samples was depleted using the QIAseq FastSelect 5S/16S/23S, rRNA Plant, rRNA Yeast, and custom rRNA Algae Depletion kits (Qiagen, Hilden, Germany) and libraries were constructed using the TruSeq Stranded mRNA kit (Illumina, San Diego, CA, USA) following standard JGI protocols. Sequencing of the flow cell was performed on the Illumina NovaSeq sequencer using NovaSeq XP V1.5 reagent kits, S4 flow cell as a 2 × 151 indexed run. Reads were quality controlled using reported JGI protocols. BBDuk (version 39.01, JGI, default parameters) was used to remove adapter sequences, remove reads with multiple “N” bases, remove reads ≤49 bp or quality score <10. Reads were mapped with BBMap (version 39.01, default parameters) (12) to select organisms including human, cat, dog, mouse, and microbial contaminants as well as ribosomal RNA species. Reads were removed that aligned with any reference sequence set with ≥93% identity. We submitted the filtered FASTQ reads to NCBI Sequence Read Archive (SRA), and raw FASTQ reads are available directly from JGI (Table 1).

**TABLE 1:** Summary of RNA-seq sample data

Sample	Strain	Carbon source(s)	Replicate number	Total filtered paired reads	SRA accession number	Raw FASTQ JGI file name
NG_1	Parent	Glucose	1	8,261,114	SRX27744256	52934.3.507152.AAGTCGAGAT-AAGTCGAGG.fastq.gz
NG_2	Parent	Glucose	2	7,998,812	SRX27744257	52934.3.507152.GGACTAGAAT-GGACTAGAG.fastq.gz
NPG_1	Parent	PCA + glucose	1	6,902,516	SRX27744258	52934.3.507152.CTCATCAGAT-CTCATCAGG.fastq.gz
NPG_2	Parent	PCA + glucose	2	7,400,166	SRX27744259	52934.3.507152.TCGTAGTCAT-TCGTAGTCG.fastq.gz
N4G_1	Parent	4-CA + glucose	1	8,765,942	SRX27744260	52934.3.507152.ACCTTCTCAT-ACCTTCTCG.fastq.gz
N4G_2	Parent	4-CA + glucose	2	11,861,436	SRX27744261	52934.3.507152.TGGACTCTAT-TGGACTCTG.fastq.gz
NVG_1	Parent	VA + glucose	1	11,170,954	SRX27744262	52934.3.507152.TCTAACGCAT-TCTAACGCG.fastq.gz
NVG_2	Parent	VA + glucose	2	10,620,082	SRX27744263	52934.3.507152.AGCTTGAGAT-AGCTTGAGG.fastq.gz
NSG_1	Parent	SA + glucose	1	9,324,358	SRX27744264	52934.3.507152.GGTATAGGAT-GGTATAGGG.fastq.gz
NSG_2	Parent	SA + glucose	2	12,992,624	SRX27744265	52934.3.507152.CCGGAATTAT-CCGGAATTG.fastq.gz
LG_1	Mutant	Glucose	1	10,416,308	SRX27744266	52934.3.507152.TGTTGTGGAT-TGTTGTGGG.fastq.gz
LG_2	Mutant	Glucose	2	8,867,776	SRX27744267	52934.3.507152.TCCTGCTAAT-TCCTGCTAG.fastq.gz
LPG_1	Mutant	PCA+ glucose	1	10,786,372	SRX27744268	52934.3.507152.GGAAGGATAT-GGAAGGATG.fastq.gz
LPG_2	Mutant	PCA + glucose	2	9,115,976	SRX27744269	52934.3.507152.TGGAGTTGAT-TGGAGTTGG.fastq.gz
LPG_3	Mutant	PCA + glucose	3	10,065,984	SRX27744270	52934.3.507152.CAATGTGGAT-CAATGTGGG.fastq.gz
L4G_1	Mutant	4-CA + glucose	1	10,814,906	SRX27744271	52934.3.507152.CGAAGAACAT-CGAAGAACG.fastq.gz
L4G_2	Mutant	4-CA + glucose	2	11,394,736	SRX27744272	52934.3.507152.GACGATCTAT-GACGATCTG.fastq.gz
L4G_3	Mutant	4-CA + glucose	3	9,511,994	SRX27744273	52934.3.507152.AGATGAGGAT-AGATGAGGG.fastq.gz
LVG_1	Mutant	VA + glucose	1	9,905,952	SRX27744274	52934.3.507152.ATGACCAGAT-ATGACCAGG.fastq.gz
LVG_2	Mutant	VA + glucose	2	9,338,470	SRX27744275	52934.3.507152.ATCGGTGTAT-ATCGGTGTG.fastq.gz
LVG_3	Mutant	VA + glucose	3	12,642,560	SRX27744276	52934.3.507152.CTGGTTCTAT-CTGGTTCTG.fastq.gz
LSG_1	Mutant	SA + glucose	1	11,232,974	SRX27744277	52934.3.507152.TCCGTATGAT-TCCGTATGG.fastq.gz
LSG_2	Mutant	SA + glucose	2	9,211,142	SRX27744278	52934.3.507152.GGATCTTCAT-GGATCTTCG.fastq.gz
LSG_3	Mutant	SA + glucose	3	8,357,162	SRX27744279	52934.3.507152.TGATACGCAT-TGATACGCG.fastq.gz

RNA-seq data sets have proven useful for studying metabolism in *N. aromaticivorans* (11). The reported transcriptomic data sets will be useful to decipher the regulatory network associated with the *N. aromaticivorans* LysR-type TF SARO_RS14285.

## Data Availability

Filtered RNA-seq FASTQ files are available from NCBI SRA, BioProject Accession Number PRJNA1226177). Raw RNA-seq FASTQ files and other sequencing files are available for download from the JGI Data Portal (https://data.jgi.doe.gov/, Proposal ID 510025).
